# Fumonisin B1-Induced Changes in Cotton Fiber Elongation Revealed by Sphingolipidomics and Proteomics

**DOI:** 10.3390/biom10091258

**Published:** 2020-08-31

**Authors:** Li Wang, Chen Liu, Yujie Liu, Ming Luo

**Affiliations:** 1 Zhengzhou Research Base, State Key Laboratory of Cotton Biology, Zhengzhou University, Zhengzhou 450001, China; wangli07-2@163.com (L.W.); liuchen_lc@163.com (C.L.); liuyujie19@126.com (Y.L.); 2 State Key Laboratory of Cotton Biology, Institute of Cotton Research, Chinese Academy of Agricultural Sciences, Anyang 455000, China; 3 Key Laboratory of Biotechnology and Crop Quality Improvement of Ministry of Agriculture, Biotechnology Research Center, Southwest University, Chongqing 400716, China

**Keywords:** cotton, fiber elongation, fumonisin B1, sphingolipid, proteomic

## Abstract

Sphingolipids are essential biomolecules and membrane components, but their regulatory role in cotton fiber development is poorly understood. Here, we found that fumonisin B1 (FB1)—a sphingolipid synthesis inhibitor—could block fiber elongation severely. Using liquid chromatography tandem mass spectrometry (LC-MS/MS), we detected 95 sphingolipids that were altered by FB1 treatment; of these, 29 (mainly simple sphingolipids) were significantly increased, while 33 (mostly complex sphingolipids) were significantly decreased. A quantitative analysis of the global proteome, using an integrated quantitative approach with tandem mass tag (TMT) labeling and LC-MS/MS, indicated the upregulation of 633 and the downregulation of 672 proteins after FB1 treatment. Most differentially expressed proteins (DEPs) were involved in processes related to phenylpropanoid and flavonoid biosynthesis. In addition, up to 20 peroxidases (POD) were found to be upregulated, and POD activity was also increased by the inhibitor. To our knowledge, this is the first report on the effects of FB1 treatment on cotton fiber and ovule sphingolipidomics and proteomics. Our findings provide target metabolites and biological pathways for cotton fiber improvement.

## 1. Introduction

Cotton is the world’s leading fiber crop, and cotton fibers are unicellular, linear extensions of the seed epidermis. Fiber cell development in cotton can be divided into four distinctive but overlapping stages: fiber initiation, cell elongation, secondary cell wall deposition, and fiber maturation [1]. Cell elongation is important for the growth and development of fiber cells, and for cotton fiber quality [2,3]. However, the molecular mechanism regulating fiber elongation is still poorly understood.

As cotton fibers are extremely elongated single cells, to meet the structural needs of cell elongation and to regulate the orderly increase in various metabolic activities during the elongation process, besides membrane expansion—which includes both the cytoplasmic and inner membranes—changes in the corresponding membrane properties are also required. Therefore, membranes play important roles in the growth and development of cotton fiber [4]. Sphingolipids and sterols are important components of cell membranes, and play important roles in the regulation of membrane fluidity, permeability, and membrane-binding protein activity [5,6,7]. As phytosterols (especially sitosterol) have higher fiber elongation stage and lower fiber secondary cell wall deposition stage, changes in sterol content and composition affect fiber cell development [8]. Although phytosterols play important roles in fiber development, the role of sphingolipids in this process is not clear.

Sphingolipids are widely found in eukaryotes and in a few prokaryotic membranes [9]. Having complex and diverse structures [10] not only they are the main structural components of membranes, but they are also important bioactive molecules, and are involved in various signal transduction pathways, including programmed cell death (PCD) [11,12,13], hypersensitivity induced by pathogens [11,14,15], ABA-dependent guard cell closure [16,17,18], host-pathogen interaction [19] and low temperature signal transduction [20,21].

Sphingolipid synthesis inhibitors, such as FB1 and PDMP, are important for understanding sphingolipid function [22,23]. FB1 is a water-soluble metabolite produced by *Fusarium moniliforme* and has a structure very similar to that of sphinganine (Sph) [24]. As a specific inhibitor of the ceramide synthase pathway, the effect of FB1 on sphingolipid synthesis and its applications have been widely studied [25,26,27]. Here, we show that FB1 could severely block cotton fiber elongation. Furthermore, we performed sphingolipidomic and proteomic analyses on the cotton fibers and ovules, to identify key sphingolipids and biological pathways in cotton fiber elongation. Our results provide new insights into the regulatory mechanisms behind cotton fiber elongation.

## 2. Materials and Methods

### 2.1. Cotton Materials and In Vitro Ovule Culture

Upland cotton (*Gossypium hirsutum* L.) plants were grown under natural field conditions in Zhengzhou, Henan Province. For the in vitro ovule cultures, ovules of cotton (*Gossypium hirsutum* L.) were collected at two days post-anthesis (DPA) (blooms at anthesis were tagged and dated, and ovules were collected after two days), sterilized in 0.1% mercuric chloride solution, and cultured in Beasley and Ting’s medium [28] at 32 °C, in the presence of 1 µM FB1 (dissolved in 1 mM of dimethyl sulfoxide [DMSO] stock solution) or 0.1% DMSO (control). The control and FB1 treated sample were taken from ovules in the same boll. Ovules came from three bolls (20 ovules for each treatment) from three different cotton plants as one biological replicate. Ovule culture was performed for three consecutive days as three biological replicates. Samples were collected after 10 days of dark culture for lipid, protein and RNA extraction. Fresh samples were observed using a scanning electron microscope (SEM) (SU 3500, Hitachi, Tokyo, Japan).

### 2.2. Lipid Extraction

Freshly collected control and FB1 samples were weighed and inactivated with hot isopropanol, using a modified protocol as described previously [29]. Following inactivation, samples were extracted by chloroform:methanol: 300 mM ammonium acetate (30:41.5:3.5) (*v*/*v*/*v*), and incubated at room temperature for 24 h at 150 YPM. After incubation the samples were centrifuged, and the clear supernatants were transferred to fresh tubes. The inactivation and extraction were repeated once again, and the resulting lipid extracts from both rounds of extraction were pooled and dried in a SpeedVac (Genevac, Ipswich, UK). They were stored at −80 °C until the LC-MS analyses.

### 2.3. Lipidomics

Analyses were conducted using an Exion ultra performance liquid chromatography (UPLC) (AB Sciex, CA, USA), coupled with Sciex QTRAP 6500 PLUS (AB Sciex, CA, USA), as reported previously [30]. Lipids were separated using a Phenomenex Luna 3 μm-silica column (Phenomenex, CA, USA) (internal diameter 150 × 2.0 mm) under the following conditions: mobile phase A (chloroform: methanol: ammonium hydroxide, 89.5:10:0.5) and mobile phase B (chloroform: methanol: ammonium hydroxide: water, 55:39: 0.5:5.5). The gradient began with 95% of mobile phase A for 5 min, and was followed by a linear reduction to 60% mobile phase A over 7 min. The gradient was held for 4 min, and the mobile phase A was then further reduced to 30% and held for 15 min. MRM transitions were constructed for the comparative analysis of the various sphingolipids [31]. The individual sphingolipid classes were quantified by referencing spiked internal standards, namely, Cer d18:1/17:0, GluCer d18:1/12:0, d17:1-S1P, *D*-ribo-phytosphingosine C17, and d17:1-Sph from Avanti Polar Lipids (Alabaster, AL, USA), and GM1 d18:1/18:0-d3 from Matreya LLC (State College, PA, USA).

### 2.4. Protein Extraction and Digestion by Trypsin

Samples were powdered in liquid nitrogen using a mortar and pestle and transferred to 5 mL centrifuge tubes. Following this, four volumes of pre-cooled lysis buffer (containing 10 mM dithiothreitol [DTT], 1% protease inhibitor cocktail, 50 μM PR-619, 50 mM NAM, and 3 μM TSA) were added, and samples were sonicated on ice using a high intensity ultrasonic processor (Scientz, Ningbo, China). Samples were extracted with equal volumes of Tris-saturated phenol (pH 8.0) and centrifuged for 10 min at 5500× *g* (4 °C). The resulting supernatants were transferred to new tubes and were precipitated overnight at −20 °C by five volumes of 0.1 M ammonium acetate/methanol. Precipitates were then washed with ice-cold methanol and acetone, and proteins were re-dissolved in 8 M urea. Protein concentrations were determined by the BCA protein assay (Thermo Fisher Scientific, Rockford, IL, USA) according to the manufacturer’s instructions.

For the digestion with trypsin, the protein solution was first reduced with 5 mM DTT for 30 min at 56 °C and alkylated with 11 mM iodoacetamide for 15 min at room temperature in the dark. Samples were then diluted by adding 0.1 M TEA buffer, to obtain a urea concentration of less than 2 M. Finally, for the first overnight digestion (at 37 °C) trypsin was added at a 1:50 trypsin:protein mass ratio, and 1:100 trypsin:protein mass ratio for a second 4 h digestion.

### 2.5. Tandem Mass Tag (TMT) Labeling, HPLC Fractionation, and LC-MS/MS Analysis

For the TMT labeling, the peptides digested by trypsin were desalted using a Strata X C18 SPE column (Phenomenex, Torrance, CA, USA) and vacuum-dried. Peptides were reconstituted in 0.5 M TEA buffer and processed according to the manufacturer’s protocol for a 6-plex TMT kit (Thermo Fisher Scientific, Rockford, IL, USA). Briefly, the thawed TMT reagent was dissolved in acetonitrile, and mixed with the peptides. The mixtures were incubated for 2 h at room temperature, and then the labeled peptides were pooled, desalted, and dried by vacuum centrifugation.

For the HPLC fractionation, the labeled peptides were fractionated by high pH reverse-phase HPLC, using an Agilent 300 Extend C18 column (Agilent, Santa Clara, CA, USA) (5 μm particles, 4.6 mm ID, 250 mm length). Briefly, peptides were first separated into 60 fractions over 60 min, using a gradient of 8% to 32% acetonitrile (pH 9.0). Next, peptides were combined into 18 fractions, and dried by vacuum centrifugation.

For the LC-MS/MS analysis, peptides dissolved in 0.1% formic acid solution (solvent A) were separated using an EASY-nLC 1000 super high-performance liquid system (Thermo Fisher Scientific, Rockford, IL, USA). The gradient was comprised of an increase from 9% to 26% of solvent B (0.1% formic acid in 90% acetonitrile solution) over 23 min, 26% to 38% in 9 min, climbing to 80% in 4 min, then holding at 80% for the last 4 min, all at a constant flow rate of 500 nL/min. The separated peptides were ionized by an NSI source, followed by MS/MS in a Q Exactive^TM^ Plus (Thermo Fisher Scientific, Rockford, IL, USA) coupled online to the UPLC. The applied electrospray voltage was set at 2.0 kV. The *m*/*z* scan range was 350 to 1800 for the full scan, and the detected scan resolution of the intact peptides and fragments in the Orbitrap was 70,000 and 17,500, respectively. A data-dependent procedure that alternated between one MS scan followed by 20 MS/MS scans with 30 s dynamic exclusion was performed. Peptides were selected for MS/MS using an NCE setting of 28. Automatic gain control and fixed first mass were set as 5E4 and 100 *m*/*z*, respectively.

### 2.6. Database Search and Bioinformatic Methods

The resulting MS/MS data were processed using the MaxQuant search engine v.1.5.2.8 (https://www.maxquant.org/). Tandem mass spectra were searched against the UniProt *Gossypium hirsutum* database. Trypsin/P was set as the cleavage enzyme, and up to two missing cleavages were allowed. The mass error tolerance was set as 20 ppm (in the first search), 5 ppm in the main search for the precursor ions, and 0.02 Da for the fragment ions. Cysteine carbamidomethylation and methionine oxidation were specified as fixed and variable modifications, respectively. TMT-6plex was selected as the protein quantification method, FDR was adjusted to < 1%, and the score of peptide ion was set to > 40.

Gene Ontology (GO) annotation of the proteome was obtained from the UniProt-GOA database (https://www.ebi.ac.uk/GOA/). First, the GO ID was matched with the UniProt ID, and then the corresponding information was retrieved from the UniProt GOA database (according to the GO ID). Based on the protein sequence alignment method and the annotated domain functional description of the differentially expressed proteins (DEPs), the InterProScan software v.5.14 (http://www.ebi.ac.uk/interpro/) was used to predict those GO functional proteins that were not annotated by the UniProt-GOA database. Subsequently, the DEPs were classified by the GO annotation to three categories: biological processes, cellular components, and molecular functions. The subcellular localization of DEPs was predicted using WoLF PSORT software v.0.2 (https://www.genscript.com/wolf-psort.html).

Kyoto Encyclopedia of Genes and Genomes (KEGG) database was used to annotate the protein pathways, and KAAS v.2.0 (https://www.genome.jp/kaas-bin/kaas_main) the KEGG online service tool, was used to annotate the proteins’ KEGG database description. The annotation results were then mapped to the KEGG pathway database, using the online KEGG mapper v2.5 (http://www.kegg.jp/kegg/mapper.html).

The functional enrichment analyses (GO, KEGG pathway, and protein domain) on the DEPs were performed among the FB1 treated samples and the control. The enrichment of the DEPs against all identified proteins was examined by a two-tailed Fisher’s exact test, and the corresponding GO, pathway, or protein domain with a *p* value < 0.05 were considered significant. The GO annotation classified proteins into three categories: biological processes, cellular compartments, and molecular functions. KEGG pathways were classified into hierarchical categories according to the KEGG website.

For the enrichment-based clustering, all the obtained categories after the enrichment were collected (along with their *p* values) and those categories that were enriched at least in one of the clusters with a *p* value < 0.05, were filtered. Next, the filtered *p* value matrix was transformed using the function x = −log10 (*p* value).

### 2.7. RNA Extraction and Semi-Quantitative PCR

Total RNA from the control and the FB1 samples was extracted using the RNAprep pure Plant Kit (TIANGEN, Beijing, China). First-strand cDNAs were synthesized using the PrimeScript™ RT reagent Kit with gDNA Eraser (TAKARA, Kyoto, Japan). Semi-quantitative PCR reactions were performed with the 2xTaq Plus Master Mix (Dye Plus) (Vazyme, Nanjing, China). The PCR conditions were as follows: 95 °C for 2 min; 95 °C for 15 s, 55 °C for 20 s, 72 °C for 30 s and 28 cycles. Three biological repetitions were performed. The specific primers for the selected genes and the internal control (*Gbp* gene) are listed in Table S1.

### 2.8. Enzyme Activity Determination

Peroxidase activity was determined from approximately 0.1 g of control and FB1 samples with a Tecan 200 PRO microplate reader (TECAN, Männedorf, Switzerland) (at 470 nm), using a peroxidase activity detection kit (Solarbio, Beijing, China) according to the manufacturer’s instructions.

## 3. Results

### 3.1. FB1 Blocked the Elongation of Cotton Fibers

**Figure 1:**
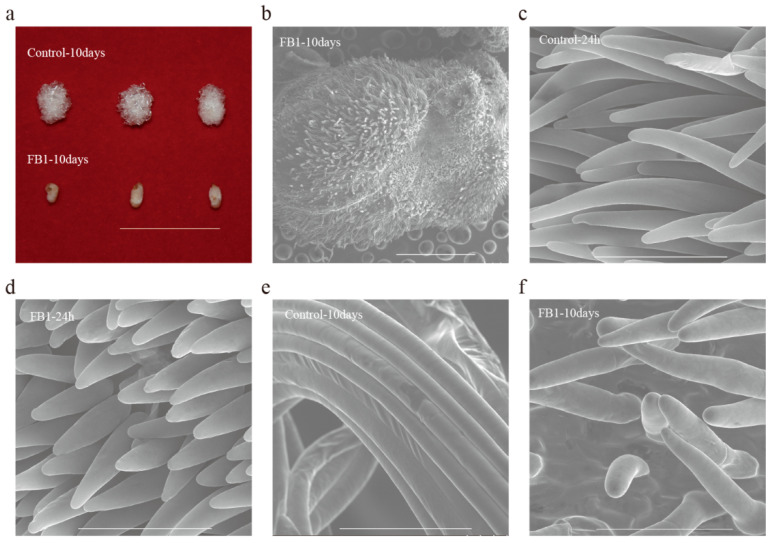
The elongation of cotton fiber is blocked by the sphingolipid synthesis inhibitor FB1. (**a**) WT ovules were collected at 2 DPA and cultured for 10 days in the culture media with DMSO or FB1, bar = 2 cm. (**b**) Scanning electron microscopy of FB1 sample cultured for 10 days, bar = 1 mm. (**c**,**d**) Scanning electron microscopy of the control (**c**) and FB1 sample (**d**), cultured for 24 h, bar = 100 μm. (**e**,**f**) Scanning electron microscopy of the control (**e**) and FB1 sample (**f**) cultured for 10 days, bar = 100 μm.

The in vitro cotton ovule culture system allows research on fiber development under controlled conditions. We used this system to study the role of sphingolipids in cotton fiber development under FB1. The elongation of cotton fibers was severely blocked by FB1 (compared with control), which was almost invisible to the naked eye, but could be seen clearly under SEM (Figure 1a,b). Thus, we further examined the elongation of cotton fibers, treated by FB1 for 24 h and 10 days, using SEM. The SEM results indicated that fiber elongation was already blocked after 24 h, and the fiber lengths from the 10 day-treated group were only slightly longer than the 24 h group (Figure 1c–f). These results revealed that the inhibition of sphingolipid biosynthesis blocked fiber cell elongation, suggesting that sphingolipids may play important role in this process.

### 3.2. Sphingolipid Homeostasis Was Disrupted by FB1 in Cotton Fibers and Ovules

**Figure 2:**
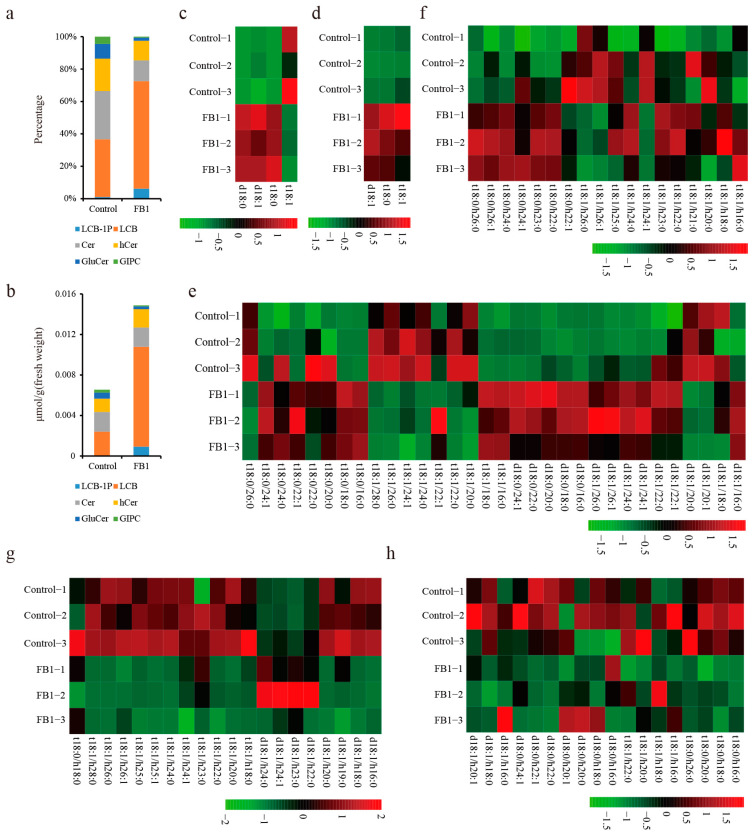
Changes in the sphingolipid content between the control and the FB1 samples. (**a**) Percentages of the six major categories of sphingolipids in the control and FB1 sample cultured 10 days. (**b**) The total content of six major sphingolipid categories in control and the FB1 sample cultured 10 days. (**c**–**h**) Heat map of the individual sphingolipids between the control and the FB1 sample cultured 10 days. c, LCB-1P; d, LCB; e, Cer; f, hCer; g, GluCer; h, GIPC.

FB1 blocks fiber cell elongation by disrupting sphingolipid metabolism. However, since the sphingolipid biosynthetic pathway is complex and thousands of unique sphingolipid structures can be found in plants, to understand which sphingolipids are important for cotton fiber development, we analyzed them by LC-MS/MS. Results showed six major categories of sphingolipids in the cotton fibers and ovules: long chain bases (LCB), long chain base-1-phosphates (LCB-1P), ceramides (Cer), hydroxyceramides (hCer), glucosylceramides (GluCer), and glycosyl inositol phosphoceramides (GIPC), with 3, 4, 32, 18, 20, and 18 lipid species, respectively (Table S2). We also calculated the total content (the sum of individual sphingolipid content in each category) and percentage (Ratio of total sphingolipids content in each category to total sphingolipids content in each sample) of sphingolipids in the control and FB1 sample (Figure 2a,b). In our study, LCBs were the most abundant (35.62%) and GIPC content was only 4.47% in cotton fibers and ovules (Figure 2a). By analyzing the content of sphingolipid species in the FB1 treatment, we found a 2.27-fold increase in the total sphingolipids compared to control. Furthermore, LCB, LCB-1P and hCer increased by 8.25, 63.49 and 1.38-fold, respectively, in the FB1 compared to the control (Figure 2b). GluCer and GIPC contents in the FB1 samples were only 46.09% and 33.65% that of the control, respectively (Figure 2b). Only Cer contents did not differ between FB1 and the control (Figure 2b). These results indicate that those sphingolipids that were above the ceramide synthesis step (including LCB and LCB-1P) increased more than 4-fold, which is possibly caused by inhibition of their consumption. At the same time, the end products of the sphingolipid synthesis pathway (including GluCer and GIPC) decreased, probably because their precursors were reduced following the FB1 application.

In order to further analyze the importance of sphingolipids for fiber development, we analyzed the individual sphingolipid contents in the FB1 samples and controls by LC-MS/MS. Among the simple sphingolipids, six LCBs were significantly increased (more than two-fold) in the FB1 sample compared to control and only LCB-1P t18:1 was slightly decreased (Figure 2c,d). All the examined Cer d18:0, with fatty acids moieties of different lengths (16:0, 18:0, 20:0, 22:0 and 24:0-FA), increased significantly in the FB1 samples (by 10.94-, 5.73-, 1.96-, 6.30- and 3.66-fold, respectively) compared to the control (Figure 2e). Cer d18:1, which contains 20:1 and 20:0-FA, decreased, but 16:0, 24:0, 24:1, and 26:0-FA increased in the FB1 samples (Figure 2e). Cers or hCers containing t18:1 and VLCFA (24:0, 24:1, 26:0, 28:0, h24:1, h26:1, and h26:0-FA) decreased, and most hCers containing saturated LCB t18:0 increased, except for the hCer t18:0/22:1 (Figure 2e,f). Complex sphingolipids, including GluCer and GIPC, decreased significantly after the FB1 treatment (Figure 2g,h). All of the detected GluCer contained unsaturated LCBs (except for GluCer t18:0/h18:0) and hydroxylated FAs (Figure 2g). Three GIPCs (t18:0/h26:0, d18:0/h22:0 and d18:0/h22:1) decreased to less than one-tenth of the control levels following the FB1 treatment (Figure 2h). Thus, the sphingolipids that differed between control and the FB1 samples, may play important roles in cotton fiber development.

### 3.3. Quantitative Proteome Analysis and the Impacts of FB1 on the Global Proteome of Cotton Fibers and Ovules

**Figure 3:**
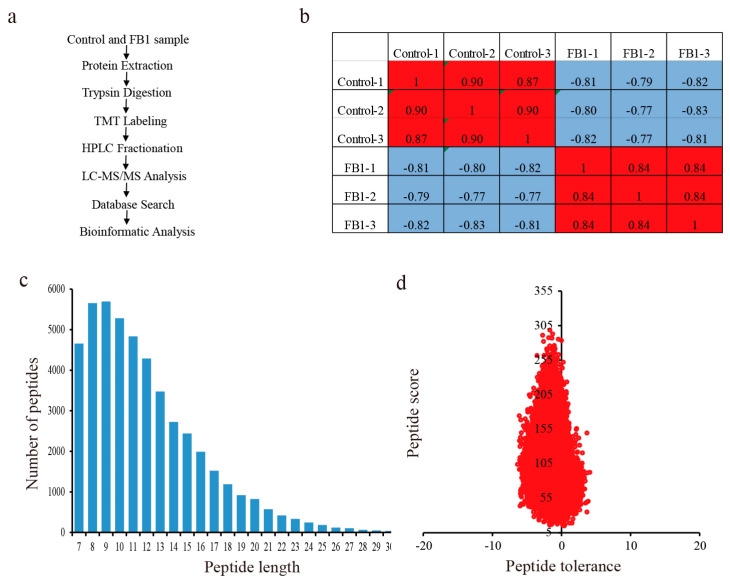
Experimental strategy for the quantitative proteome analysis and the mass spectrometry data for the quality control analysis. (**a**) Experimental strategy for the quantitative proteome analysis. (**b**) Pearson correlation coefficients from all quantified proteins between each pair of samples. (**c**) Length distribution of all identified peptides. (**d**) Peptide mass tolerance distribution.

The DEPs between FB1 and control were identified using high-throughput quantitative proteomics and TMT labeling (Figure 3a). We performed three biological replicates in both the control and the treatment groups. The heatmap of Pearson correlation coefficients from all quantified proteins between each pair of samples showed that the same treatments had high repeatability and low variance (Figure 3b). The length of most peptides varied between 7 and 16 amino acids, and most of the peptide mass errors were less than 5 ppm, meaning that the accuracy of the MS data was according to the requirements (Figure 3c,d).

**Figure 4:**
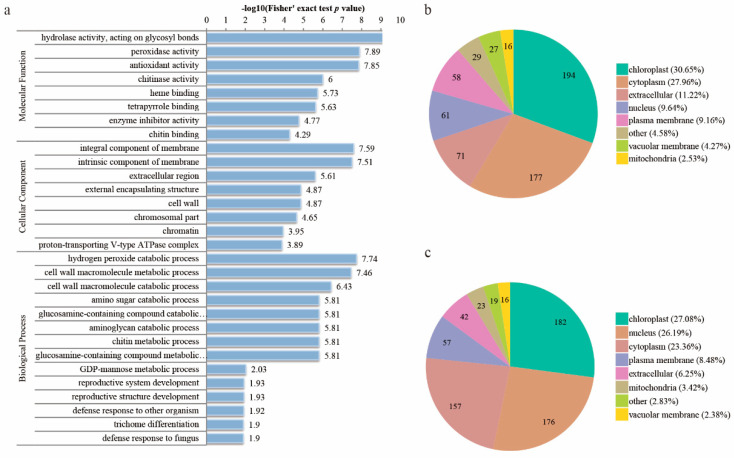
GO enrichment analysis and subcellular localization chart of DEPs in the control and the FB1 samples. (**a**) GO enrichment analysis of DEPs in control and the FB1 sample. (**b**) Subcellular localization chart of the up-regulated proteins. (**c**) Subcellular localization chart of the down-regulated proteins.

We identified a total of 9435 proteins, 7301 of which were quantified (Table S3). A quantitative FB1 vs. control ratio higher than 1.5 was considered as up-regulation, while a ratio of less than 1/1.5 was considered to be down-regulation. In this way, a total of 633 proteins were up-, and 672 proteins were down-regulated (Table S4). GO analysis (including molecular function, cellular component, and biological process) showed that the top three significantly enriched molecular function GO terms of the DEPs were: hydrolase activity (−log10 (*p* value) = 9.43), peroxidase activity (−log10 (*p* value) = 7.89), and antioxidant activity (−log10 (*p* value) = 7.85). The cellular component GO terms of the DEPs were mainly associated with terms that included integral component of membrane (−log10 (*p* value) = 7.59), intrinsic component of membrane (−log10 (*p* value) = 7.51), and extracellular region (−log10 (*p* value) = 5.61). The main enriched biological process GO terms of the DEPs were associated with the hydrogen peroxide catabolic process (−log10 (*p* value) = 7.74), cell wall macromolecule metabolic process (−log10 (*p* value) = 7.46), and the cell wall macromolecule catabolic process (−log10 (*p* value) = 6.43) (Figure 4a).

We also performed subcellular predictions to characterize the subcellular localization of DEPs using WoLF PSORT software. As shown in (Figure 4b,c), most of the up-regulated proteins were localized in the chloroplast (30.65%), cytoplasm (27.96%), and the extracellular region (11.22%), while the majority of the down-regulated proteins were distributed in the chloroplast (27.08%), nucleus (26.19%), and cytoplasm (23.36%)(Figure 4b,c). The percentage of proteins located in the plasma membrane was similar between the up- and down-regulated proteins. The percentage of down-regulated proteins in the nucleus and mitochondria was higher than that of the up-regulated ones, while the percentage of up-regulated proteins, localized in the chloroplast, cytoplasm, extracellular region and vacuolar membrane, was higher than that of the down-regulated ones.

### 3.4. Enrichment Analysis of the DEPs in Cotton Fiber and Ovule under FB1

**Figure 5:**
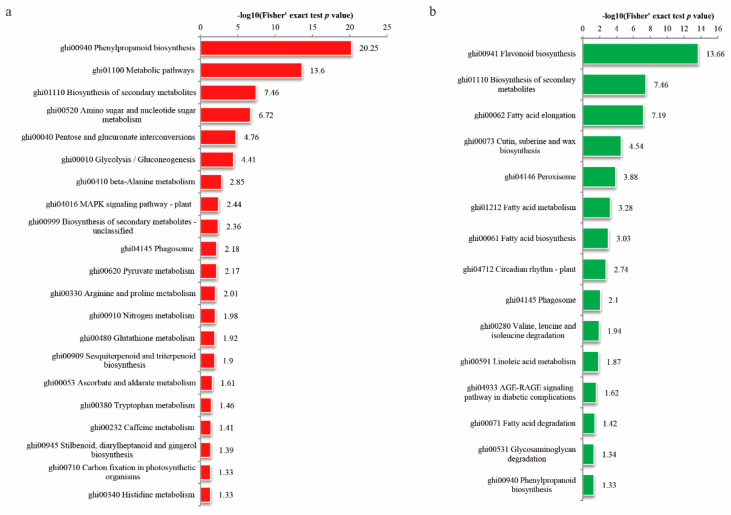
KEGG enrichment analysis of cotton fibers and ovules treated by FB1 and the control. (**a**) Up-regulated proteins (red bars); (**b**) down-regulated proteins (green bars).

To further understand the DEPs in the cotton fibers and ovules as result of the FB1 treatment, functional enrichment analyses (including KEGG pathway and protein domain) were performed. KEGG pathway analysis revealed that the highly enriched, upregulated DEPs were connected with phenylpropanoid biosynthesis (−log10 (*p* value) = 20.25), metabolic pathways (−log10 (*p* value) = 13.6), biosynthesis of secondary metabolites (−log10 (*p* value) = 7.46), and amino sugar and nucleotide sugar metabolism (−log10 (*p* value) = 6.72) (Figure 5a). The downregulated proteins were mostly involved in flavonoid biosynthesis (−log10 (*p* value) = 13.66), the biosynthesis of secondary metabolites (−log10 (*p* value) = 7.46), and fatty acid elongation (−log10 (*p* value) = 7.19) (Figure 5b).

Domain enrichment analysis of the DEPs showed that many of the up-regulated proteins contained the following domains: secretory peroxidase; heme peroxidase, plant/fungal/bacterial; plant methyltransferase dimerization; *O*-methyltransferase, family2; glycoside hydrolase, catalytic domain; berberine/berberine-like; chitin-binding, type1; glycoside hydrolase, family19, catalytic; and lysozyme-like domain (−log10 (*p* value)> 5) (Figure S1a). In contrast, the downregulated proteins mostly contained the following domains: SGNH hydrolase-type esterase domain; FAE1/type III polyketide synthase-like protein; villin headpiece; 3-oxoacyl-[acyl-carrier-protein (ACP)] synthase III, C-terminal; GDSL lipase/esterase; calcium permeable stress-gated cation channel 1, N-terminal transmembrane domain; calcium-dependent channel, 7 TM region, putative phosphate; 10TM putative phosphate transporter, cytosolic domain; thiolase-like, linker histone H1/H5, domain H15; zinc finger, CCHC-type; ABC-2 type transporter; remorin, C-terminal (−log10 (*p* value)> 4) (Figure S1b).

### 3.5. FB1 Altered the Phenylpropanoid Biosynthesis Pathway in Cotton Fibers and Ovules

**Figure 6:**
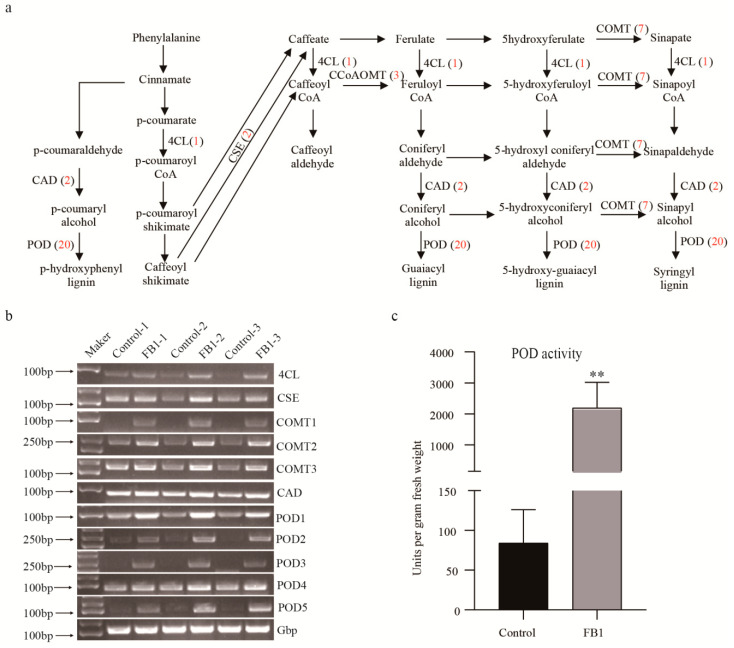
Involvement of the phenylpropanoid biosynthesis [33] in cotton fibers and ovules treated by FB1. (**a**) Up-regulated proteins from the phenylpropanoid biosynthesis pathway. The numbers in red represent proteins up regulated at the FB1 treated samples. (**b**) Semi-quantitative PCR of the phenylpropanoid biosynthesis-related genes in control and the FB1 sample. DL2000 DNA marker (left). Amplified genes of interest (right). (**c**) POD activity of control and the FB1 sample. Double asterisks indicate statistically significant differences between FB1 treatment and the control, as determined by Student’s t-test (**, *p* < 0.01).

The KEGG pathway analysis showed that proteins from the phenylpropanoid biosynthesis pathway were the most significantly upregulated. In total, 38 up-regulated phenylpropanoid biosynthesis-related proteins were identified (Figure 6a, Table S2). From these, 20 were peroxidases (POD), 7 caffeic acid 3-*O*-methyltransferases (COMT), 3 caffeoyl CoA *O*-methyltransferases (CCoAOMT), 2 caffeoylshikimate esterases (CSE), 2 cinnamyl alcohol dehydrogenases (CAD), one feruloyl CoA ortho-hydroxylase (F6′H), one cannabidiolic acid synthase (CBDAS), one 4-coumarate-CoA ligase (4CL) and one aldehyde dehydrogenase (ALDH). Thereafter, we randomly selected genes coding for 5 *PODs*, 3 *COMTs*, and one *4CL*, *CSE,* and *CAD* each, to examine changes in their transcription levels by semi-quantitative PCR. Our results showed that the transcription of each gene was consistent with the changes detected in their protein levels (Figure 6b). Similarly, to confirm the results of the proteome profiling, we measured the peroxidase activity in both the control and the FB1 samples (Figure 6c). These values were significantly higher in the latter, which was consistent with the results of our proteome profiling.

## 4. Discussion

Cotton is the most important natural fiber crop worldwide. Sphingolipids are both important cell membrane components and signaling molecules. Since fiber elongation in cotton requires constant changes in both the membrane area and in the cotton fiber properties—processes where sphingolipids may play important roles—understanding the molecular mechanisms involved in cotton fiber elongation through sphingolipidomics and proteomics, are of great importance.

Sphingolipids are characterized by a long-chain base sphingoid backbone with an amide-bound fatty acyl chain. Their structural diversities arise from variations in the lipid head group (simple or branched sugar residues, or neutral or charged moieties) and sphingoid base/fatty acyl chain (length, degree of saturation, methylation). Therefore, these variations produce thousands of unique sphingolipid structures [32]. Sphingolipid composition varies from species to species and organ to organ and is different depending on the developmental stage. So far, the composition and function of sphingolipids in cotton is still unknown.

In our study, six major classes of sphingolipids, containing a total of 95 sphingolipid molecules, were detected in the cotton fibers and ovules. The most abundant sphingolipids were the LCBs (35.62%) (Figure 2a), while their percentage in *Arabidopsis* seedlings was only reported to be 8.44% [30]. Conversely, the GIPC content—the largest in *Arabidopsis* seedlings (39.27%) [30]—was only 4.47% in cotton fibers and ovules (Figure 2a). Interestingly, the pattern of sphingolipid composition in cotton differed from the *Arabidopsis* seedlings, likely because cotton fibers are extremely elongated cells that start from the ovules, and this cell type is completely different from the cells of the *Arabidopsis* seedlings. The unique pattern of sphingolipid composition of the cotton fibers and ovules might also be associated with the rapid elongation of fiber cells. FB1 treatment resulted in the significant increase of 29, and the decrease of 33 sphingolipids (Figure 2). Moreover, this treatment completely disturbed the sphingolipid homeostasis in both fibers and ovules. Although total hCer content only increased by 1.38-fold—while the Cer did not change at all—the content of individual Cer and hCer sphingolipids fluctuated greatly, particularly that of Cers or hCers containing t18:1 and VLCFA (24:0, 24:1, 26:0, 28:0, h24:1, h26:1, and h26:0-FA). Almost all complex sphingolipids (e.g., GluCer and GIPC) decreased significantly after the FB1 treatment (Figure 2). These suggest that the FB1-sensitive ceramide synthase mainly mediated the synthesis of Cer and hCer by VLCFAs and t18:1, so only this part of ceramide could further synthesize GIPC and GluCer. Thus, the inhibitory effect of FB1 on the growth of fiber cells may have had two aspects: a decrease in the GIPC and GluCer contents, and the increase in the LCB and LCB-1P contents. LCB and LCB-1P accumulation has been reported to cause programmed cell death in plants [34], so it could have inhibited the growth of fiber cells. However, considering that fiber elongation can also be inhibited by the VLCFA synthase inhibitor and can be promoted by the exogenous application of C24:0 or C26:0 FA [35] we speculate that the inhibitory effect of FB1 on fiber cells would mainly be caused by the decrease in GIPC and GluCer, rather than by the increase in simple sphingolipids.

The continuous development of proteomic tools enable us more thorough studies on cotton fiber development. At the early stage of development (10 DPA), one study identified 104 proteins in cotton ovules by two-dimensional gel electrophoresis, 93 of which preferentially accumulated in the wild type, and 11 in the *fuzzless-lint-less* mutant [36]. Another study identified 6990 proteins, 336 of which were defined as DEPs between the fibers of wild versus domesticated cotton, based on the isobaric tags for relative and absolute protein quantification (iTRAQ) proteomic methods [37]. Here, using TMT-label proteomic methods, we identified 9435 proteins, 633 of which were significantly up- and 672 were significantly down-regulated in the cotton fibers blocked by FB1 (Tables S3and S4). Our study stands out through the identification of a higher number of proteins and more DEPs in the fibrous differential materials, so it provides a more comprehensive understanding of the fiber development mechanisms.

Previous studies have shown that the phenylpropanoid and flavonoid secondary metabolism pathways play important roles in the development of cotton fibers [37,38,39]. Indeed, during the developmental transitions of the cotton fiber (e.g., from elongation to secondary cell wall biosynthesis) important proteins, involved in phenylpropanoid and flavonoid secondary metabolisms, are known to be down-regulated [38]. A previous proteomic study showed alterations in the phenylpropanoid biosynthesis between fibers of the wild, versus domesticated cotton, and it showed that POD (peroxidase) activity has a great potential in fiber elongation [37]. The silencing of F3H (flavanone 3-hydroxylase), a flavonoid pathway enzyme, significantly increased the naringenin (NAR) content of fiber cells, and retarded fiber growth [39]. In this study, we identified 38 up-regulated phenylpropanoid biosynthesis proteins. Moreover, up to 20 POD proteins were found to be upregulated in the FB1 sample, together with a 26-fold increase in the POD activity, compared to the control (Figure 6). Thus, these proteins may be important targets for cotton fiber improvement, along with the regulatory roles played by sphingolipids in phenylpropanoid and flavonoid biosynthesis. In summary, studies into the relationships between sphingolipids, phenylpropanoids and flavonoids, and their roles in the cotton fiber development would be valuable in the future.

Our KEGG pathway enrichment analysis showed that four out of the 15 down-regulated pathways were associated with fatty acids (elongation, metabolism, synthesis, and degradation). A previous study showed that saturated VLCFA promotes cotton fiber elongation by activating ethylene biosynthesis, and that the fatty acid elongation pathway is involved in the VLCFA synthesis [35]. Thus, the synthesis of VLCFAs, as sphingolipid components, may be regulated by the feedback of sphingolipids. FB1 treatment resulted in significantly decreased Cers and hCers, with significantly decreased VLCFAs (24:0, 24:1, 26:0, 28:0, h24:1, h26:1, and h26:0). Thus, we could confirm that the FB1 treatment suppresses VLCFA biosynthesis.

## 5. Conclusions

In conclusion, our sphingolipidomic and quantitative proteomic study provides a comprehensive metabolic signature of the cotton fibers under the effects of FB1. From the many identified sphingolipid species, most were altered by FB1 in the cotton fibers and ovules. Our KEGG analysis showed that proteins related to the phenylpropanoid biosynthesis pathway were significantly upregulated and that POD activity was increased by FB1. Together, our results revealed key sphingolipids and pathways involved in cotton fiber elongation.

## Supplementary Materials

The following are available online at https://www.mdpi.com/2218-273X/10/9/1258/s1, **Figure S1**: Protein domain enrichment analysis of the DEPs in the control and the FB1 samples; Table S1: Primer sequences for semi-quantitative PCR; Table S2: Individual sphingolipid content of control and the FB1 sample; Table S3: The list of all proteins identified using LC-MS/MS in control and the FB1 samples; and Table S4: The list of all DEPs in the control and the FB1 samples.

## References

[1] Kim H.J., Triplett B.A. (2001). Cotton fiber growth in planta and in vitro. Models for plant cell elongation and cell wall biogenesis. Plant. Physiol..

[2] Zhao B., Cao J.-F., Hu G., Chen Z., Wang L.-Y., Shangguan X.-X., Wang L.-J., Mao Y.-B., Zhang T.-Z., Wendel J.F. (2018). Core cis-element variation confers subgenome-biased expression of a transcription factor that functions in cotton fiber elongation. New Phytol..

[3] Hernandez-Gomez M.C., Runavot J.-L., Meulewaeter F., Knox P. (2017). Developmental features of cotton fibre middle lamellae in relation to cell adhesion and cell detachment in cultivars with distinct fibre qualities. BMC Plant Boil..

[4] Xu F., Suo X., Li F., Bao C., He S., Huang L., Luo M. (2020). Membrane lipid raft organization during cotton fiber development. J. Cotton Res..

[5] Chao D.-Y., Gable K., Chen M., Baxter I., Dietrich C.R., Cahoon E.B., Guerinot M.L., Lahner B., Lü S., Markham J.E. (2011). Sphingolipids in the root play an important role in regulating the leaf ionome in Arabidopsis thaliana. Plant Cell.

[6] Sperling P., Franke S., Lüthje S., Heinz E. (2005). Are glucocerebrosides the predominant sphingolipids in plant plasma membranes?. Plant Physiol. Biochem..

[7] Tjellström H., Hellgren L.I., Wieslander Å., Sandelius A.S. (2009). Lipid asymmetry in plant plasma membranes: Phosphate deficiency-induced phospholipid replacement is restricted to the cytosolic leaflet. FASEB J..

[8] Niu Q., Tan K., Zang Z., Xiao Z., Chen K., Hu M., Luo M. (2019). Modification of phytosterol composition influences cotton fiber cell elongation and secondary cell wall deposition. BMC Plant Boil..

[9] Heaver S.L., Johnson E.L., Ley R. (2018). Sphingolipids in host–microbial interactions. Curr. Opin. Microbiol..

[10] Marquês J.T., Marinho H.S., De Almeida R.F.M., De Almeida R.F.M. (2018). Sphingolipid hydroxylation in mammals, yeast and plants—An integrated view. Prog. Lipid Res..

[11] Liang H., Yao N., Song J.T., Luo S., Lü H., Greenberg J.T. (2003). Ceramides modulate programmed cell death in plants. Genes Dev..

[12] Shi L., Bielawski J., Mu J., Dong H., Teng C., Zhang J., Yang X., Tomishige N., Hanada K., A Hannun Y. (2007). Involvement of sphingoid bases in mediating reactive oxygen intermediate production and programmed cell death in Arabidopsis. Cell Res..

[13] Ormancey M., Thuleau P., Van Der Hoorn R.A.L., Grat S., Testard A., Kamal K.Y., Boudsocq M., Cotelle V., Mazars C. (2019). Sphingolipid-induced cell death in Arabidopsis is negatively regulated by the papain-like cysteine protease RD21. Plant Sci..

[14] Wang W.-M., Yang X., Tangchaiburana S., Ndeh R., Markham J.E., Tsegaye Y., Dunn T.M., Wang G.-L., Bellizzi M., Parsons J.F. (2008). An inositolphosphorylceramide synthase is involved in regulation of plant programmed cell death associated with defense in Arabidopsis. Plant Cell.

[15] Gan Y., Zhang L., Zhang Z., Dong S., Li J., Wang Y., Zheng X.-B. (2008). The LCB2subunit of the sphingolip biosynthesis enzyme serine palmitoyltransferase can function as an attenuator of the hypersensitive response and Bax-induced cell death. New Phytol..

[16] Coursol S., Fan L.-M., Le Stunff H., Spiegel S., Gilroy S., Assmann S.M. (2003). Sphingolipid signalling in Arabidopsis guard cells involves heterotrimeric G proteins. Nature.

[17] Ng C.K.-Y., Carr K., McAinsh M.R., Powell B., Hetherington A.M. (2001). Drought-induced guard cell signal transduction involves sphingosine-1-phosphate. Nature.

[18] Worrall D., Liang Y.-K., Alvarez S., Holroyd G.H., Spiegel S., Panagopulos M., Gray J.E., Hetherington A.M. (2008). Involvement of sphingosine kinase in plant cell signalling. Plant J..

[19] Peer M., Stegmann M., Mueller M.J., Waller F. (2010). Pseudomonas syringae infection triggers de novo synthesis of phytosphingosine from sphinganine in Arabidopsis thaliana. FEBS Lett..

[20] Dutilleul C., Benhassaine-Kesri G., Demandre C., Rézé N., Launay A., Pelletier S., Renou J.-P., Zachowski A., Baudouin E., Guillas I. (2012). Phytosphingosine-phosphate is a signal for AtMPK6 activation and Arabidopsis response to chilling. New Phytol..

[21] Cantrel C., Vazquez T., Puyaubert J., Rézé N., Lesch M., Kaiser W.M., Dutilleul C., Guillas I., Zachowski A., Baudouin E. (2010). Nitric oxide participates in cold-responsive phosphosphingolipid formation and gene expression in Arabidopsis thaliana. New Phytol..

[22] Krüger F., Krebs M., Viotti C., Langhans M., Schumacher K., Robinson D.G. (2012). PDMP induces rapid changes in vacuole morphology in Arabidopsis root cells. J. Exp. Bot..

[23] Wu J.-X., Li J., Liu Z., Yin J., Chang Z.-Y., Rong C., Wu J.-L., Bi F.-C., Yao N. (2015). The Arabidopsis ceramidase AtACER functions in disease resistance and salt tolerance. Plant J..

[24] Abbas H.K., Tanaka T., Duke S.O., Porter J.K., Wray E.M., Hodges L., Sessions A.E., Wang E., Merrill A.H., Riley R.T. (1994). Fumonisin- and AAL-toxin-induced disruption of sphingolipid metabolism with accumulation of free sphingoid bases. Plant Physiol..

[25] Luttgeharm K.D., Cahoon E.B., Markham J.E. (2016). Substrate specificity, kinetic properties and inhibition by fumonisin B1 of ceramide synthase isoforms from Arabidopsis. Biochem. J..

[26] Stone J.M., Heard J.E., Asai T., Ausubel F.M. (2000). Simulation of fungal-mediated cell death by fumonisin B1 and selection of fumonisin B1-resistant (fbr) Arabidopsis mutants. Plant Cell.

[27] Stone J.M., Liang X., Nekl E.R., Stiers J.J. (2005). Arabidopsis AtSPL14, a plant-specific SBP-domain transcription factor, participates in plant development and sensitivity to fumonisin B1. Plant J..

[28] Beasley C.A., Ting I.P.J.A.j.b. (1973). The effects of plant growth substances on in vitro fiber development from fertilized cotton ovules. Am. J. Bot..

[29] Welti R. (2002). Profiling Membrane Lipids in Plant Stress Responses. ROLE OF PHOSPHOLIPASE Dalpha IN FREEZING-INDUCED LIPID CHANGES IN ARABIDOPSIS. J. Boil. Chem..

[30] Huang D.Q., Sun Y.B., Ma Z.M., Ke M.Y., Cui Y., Chen Z.C., Chen C.F., Ji C.Y., Tran T.M., Yang L. (2020). Salicylic acid-mediated plasmodesmal closure via Remorin-dependent lipid organization. Proc. Natl. Acad. Sci. USA.

[31] Markham J.E., Jaworski J.G. (2007). Rapid measurement of sphingolipids from *Arabidopsis thaliana* by reversed-phase high-performance liquid chromatography coupled to electrospray ionization tandem mass spectrometry. Rapid Commun. Mass Spectrom..

[32] Sud M., Fahy E., Cotter D., Brown A., A Dennis E., Glass C.K., Merrill A.H., Murphy R.C., Raetz C.R.H., Russell D.W. (2007). LMSD: LIPID MAPS structure database. Nucleic Acids Res..

[33] Yadav V., Wang Z., Wei C., Amo A., Ahmad B., Yang X., Zhang X. (2020). Phenylpropanoid pathway engineering: An emerging approach towards plant defense. Pathogens.

[34] Sã¡nchez-Rangel D., Vicente M.R.-S., De La Torre-Hernã¡ndez M.E., Nã¡jera-Martã-nez M., Plasencia J., Nájera-Martínez M. (2015). Deciphering the link between salicylic acid signaling and sphingolipid metabolism. Front. Plant Sci..

[35] Qin Y.-M., Hu C.-Y., Pang Y., Kastaniotis A.J., Hiltunen J.K., Zhu Y. (2007). Saturated very-long-chain fatty acids promote cotton fiber and Arabidopsis cell elongation by activating ethylene biosynthesis. Plant Cell.

[36] Pang C.-Y., Wang H., Pang Y., Xu C., Jiao Y., Qin Y.-M., Western T.L., Yu S., Zhu Y. (2010). Comparative proteomics indicates that biosynthesis of pectic precursors is important for cotton fiber and *Arabidopsis* root hair elongation. Mol. Cell. Proteom..

[37] Qin Y., Wei H., Sun H., Hao P., Wang H., Su J., Yu S. (2017). Proteomic analysis of differences in fiber development between wild and cultivated Gossypium hirsutum L.. J. Proteome Res..

[38] Zhou X., Hu W., Li B., Yang Y., Zhang Y., Thow K., Fan L., Qu Y. (2019). Proteomic profiling of cotton fiber developmental transition from cell elongation to secondary wall deposition. Acta Biochim. Biophys. Sin. (Shanghai).

[39] Tan J., Tu L., Deng F., Hu H., Nie Y., Zhang X. (2013). A genetic and metabolic analysis revealed that cotton fiber cell development was retarded by flavonoid naringenin. Plant Physiol..

