# Supplementary material for: Fumonisin B1-Induced Changes in Cotton Fiber Elongation Revealed by Sphingolipidomics and Proteomics
Source: Biomolecules. 2020 Aug 31;10(9):1258. doi: 10.3390/biom10091258 (PMC7564794; doi:10.3390/biom10091258)
Supplement: Supplementary file 1 [file biomolecules-10-01258-s001.zip › Figure S1.pdf]

Supplementary Material

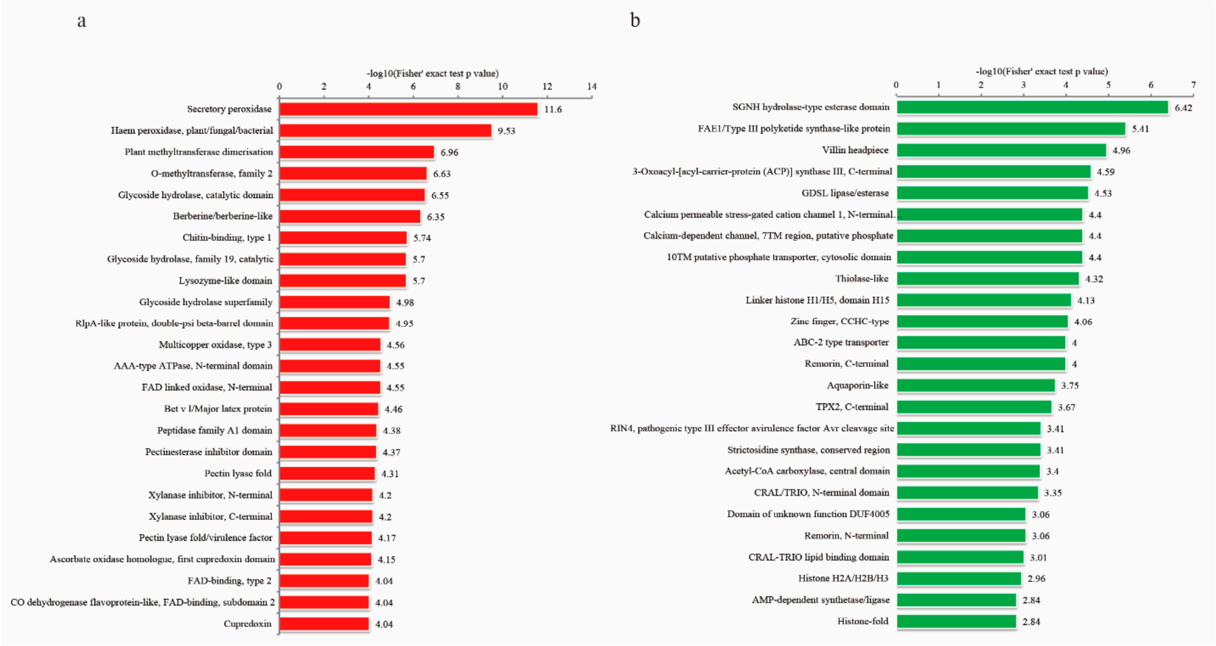

Figure S1. Protein domain enrichment analysis of the DEPs in the control and the FB1 samples. (a) Up-regulated proteins (red bars); (b) Down-regulated proteins (green bars).
